# Blast resistance of re-entrant auxetic cored sandwich panels with tunable stiffness

**DOI:** 10.1038/s41598-025-17295-5

**Published:** 2025-10-15

**Authors:** Yuanhao Zhao, Tingting Zhang, Ruonan Li, Wenjiao Zhang, Dongzhou Jia

**Affiliations:** School of Civil Engineering, Liaoning University of Technology, Jinzhou, 121001 China

**Keywords:** Auxetic, Negative Poisson’s ratio, Variable stiffness factor, Mechanical properties, Blast loading, Sandwich structure, Engineering, Materials science

## Abstract

As the threat of explosion incidents continues to intensify, it poses a significant risk to human safety. This study proposes a variable stiffness re-entrant (VSRE) core sandwich panels with negative Poisson’s ratio to enhance the explosion protection effect. Numerical simulation methods are used to analyze the impact of explosion loads on the structure, and the model is verified using the experimental results from existing studies. The VSRE core demonstrates excellent explosion protection performance. Compared with the traditional re-entrant (RE) core of equivalent mass, in the numerical simulation, the lower plate maximum displacement (*maxD*) is reduced by 11.85%, while the energy absorption (*EA*) of the system is increased by 14.39%. The variable stiffness factor of this design enables the control of density deformation, thereby achieving adjustable energy absorption capacity. The parameter study of the system shows that through the reasonable combination of core thickness, side distance, and gradient configuration, the best performance can be achieved. Specifically, combining the VSRE core with a thicker rear plate can more effectively distribute stress and material flow, reducing the peak deformation by up to 81.11%. This structure enables more efficient material flow towards the deformation center, effectively reducing the overall deformation and improving the energy absorption capacity. Therefore, the application of this variable stiffness helical plate in explosion protection systems has great potential.

## Introduction

With the increasing strike precision and destructive power of modern missiles, extensive research in the military and defense engineering fields has focused on enhancing the blast resistance of load-bearing structures. Among the various protective systems explored, sandwich panels have gained significant attention due to their lightweight nature, outstanding shear load-bearing capacity, and superior energy absorption capacity under blast loading. These structures are widely used in engineering applications such as bridges and protective infrastructures including military and defense systems^1,2^. Sandwich panels are formed by bonding two rigid facing layers to a thick, light core material. This arrangement provides better structural efficiency than solid plates at equivalent weights, synergistically combining the faces’ stiffness with the core’s deformability and mass advantages^3^. A wide variety of core designs have been developed, ranging from conventional honeycomb cores with their characteristic hexagonal cellular structure^4,5^, to waveform cores featuring corrugated plates for enhanced shear resistance^6–8^, and diamond cell cores composed of interconnected diamond-shaped units^9^. Advanced designs include lattice cores with periodic truss architectures^10,11^, bionic hybrid cores that draw inspiration from nature’s structural optimization principles to achieve synergistic combinations of strength, toughness and functional performance^12,13^, functionally graded cores with tailored density distributions^14,15^, hierarchical multi-scale cores^16^, and auxetic cores exhibiting negative Poisson’s ratio behavior^17^. Among these, honeycomb sandwich panels have been particularly well studied for their blast mitigation capabilities. Experimental studies have shown that metallic honeycomb cores provide superior attenuation of the initial blast pressure pulse compared to polystyrene or aluminum foam-filled alternatives, offering improved blast resistance^18^. Moreover, panels with thicker honeycomb cores have been found to outperform aluminum foam panels in resisting blast loads^19^. Experimental and numerical studies demonstrate that honeycomb sandwich panels exhibit smaller backside deflections than monolithic structures of equal mass. These studies also confirm that honeycomb sandwich panels provide greater energy absorption, highlighting their superior performance^20,21^. Factors influencing the explosive performance of honeycomb sandwich panels, such as core height^22^, panel material^5^, panel thickness, and cell size^23,24^, have also been investigated.

To address the limitations of conventional honeycomb cores and augment the anti-blast performance of sandwich constructions, recent research has shifted focus to alternative core structures that exhibit unconventional deformation characteristics. In this regard, auxetic honeycombs, which possess a negative Poisson’s ratio and distinctive deformation mechanisms, have emerged as a promising solution for improving the anti-explosion performance capabilities of sandwich panels. Auxetic honeycombs, characterized by a negative Poisson’s ratio (NPR) and unique deformation mechanisms, represent a promising direction for achieving enhanced blast protection capabilities. Distinct from traditional structural materials, auxetic metamaterials demonstrate NPR effect, characterized by transverse contraction under compressive loads and lateral expansion during tensile deformation. These materials demonstrate excellent shear resistance^25–27^, indentation resistance^28^, fracture resistance^29^, and energy absorption properties^30,31^. As a result, there has been significant interest in using auxetic structures as core components of sandwich panels to mitigate blast effects during extreme events^32^. Since the first fabrication of auxetic foams in 1987^33^, a variety of mesoscale and macro structured auxetic materials have been discovered from both natural and artificial sources^34^. Auxetic re-entrant honeycombs are a typical type of auxetic structure, known for being easy to design and widely applicable as a base material. Traditional NPR honeycomb metamaterials typically contain re-entrant hexagonal units, star networks, chiral structures and double arrowhead lattices. Re-entrant honeycombs, in particular, have been extensively employed as sandwich panel cores for dynamic load mitigation^35^. Imbalzano et al.^36^ conducted a comparison of blast resistance between auxetic materials and honeycomb sandwich panels, confirming that auxetic materials dissipate more energy. Additionally, Qi et al.^37^ carried out thorough experimental studies coupled with numerical simulations to examine the behavior of NPR honeycomb sandwich panels subjected to close-proximity explosions and impact scenarios. Their findings revealed that these panels exhibit significantly enhanced blast resistance characteristics compared to conventional honeycomb configurations, particularly in terms of deformation control and energy dissipation capacity. Bohara et al.^38^ investigated the transient response of NPR honeycomb configurations under near-field and far-range high-energy explosive loading conditions. Their study demonstrated these structures’ superior blast resistance, along with enhanced energy absorption and stress transfer capabilities compared to conventional honeycomb designs. Additionally, considerable research efforts have been devoted to investigating the blast mitigation capabilities of various innovative auxetic cellular configurations, including layered composites incorporating double-chevron NPR cores^39^ and functionally graded auxetic honeycomb sandwich structures^40^. Collectively, these investigations demonstrate that enhanced honeycomb cores with optimized auxetic designs can achieve superior explosion-proof capability compared to conventional structures. Nevertheless, the inherent stiffness limitations of auxetic architectures, particularly in re-entrant configurations, may compromise their protective efficacy under extreme loading conditions, where real-world applications typically require protection systems to limit back-face deformation while sustaining multiple blast events and combined blast-fragment impacts, demands that current auxetic designs often struggle to meet.

To address this challenge, extensive research efforts have been directed toward enhancing the structural stiffness of these metamaterials while preserving their NPR characteristics. For instance, Li et al.^41^ replaced the wing of the re-entrant honeycomb with a rigid rectangle to conduct various studies. Experimental results indicated that the novel cellular architecture displays pronounced scale effects in Poisson’s ratio behavior, coupled with adjustable positive/negative ratio properties, with a minimum value of – 1.05. Baran et al.^42^ enhanced in-plane structural performance of re-entrant metamaterials achieved by integrating oblique support elements into basic re-entrant unit geometries. Logacanan et al.^43^ enhanced mechanical performance by substituting the vertical elements of standard re-entrant configurations with diamond-shaped lattice units. The modified architecture demonstrated superior specific energy absorption compared to conventional re-entrant honeycomb designs. Fu et al.^44^ introduced an enhanced auxetic cellular configuration incorporating embedded rhombic elements. Computational modeling and analytical investigations revealed the modified structure’s distinctive bilinear elastic response in both stress–strain and transverse-longitudinal deformation relationships. Zhang et al.^45^ investigated an adaptive auxetic composite tubular structure with adjustable stiffness, featuring a manually adjustable densification point. Ren et al.^46^ proposed novel auxetic tubular structures by adjusting the Pattern Scale Factor (PSF), resulting in tunable mechanical properties. Cheng et al.^47^ developed re-entrant cellular architectures Variable Stiffness Factor (VSF) to enable tunable mechanical performance through controlled densification strain modulation. Experimental and numerical analysis verified the accuracy between design results and actual VSF. This validation demonstrated that the re-entrant structure’s compression point can be quantitatively tuned using the defined VSF. Moreover, the modified honeycomb configuration demonstrated significantly superior specific energy absorption performance relative to conventional re-entrant cellular architectures. In addition to improving stiffness through structural modifications, functionally graded auxetic honeycomb cores have shown superior energy absorption compared to uniform-core designs^48^. Jiang et al.^49^ developed a graded re-entrant circular auxetic core and systematically evaluated its blast mitigation performance through multi-objective optimization analysis. The results show that the lower plate maximum displacement (*maxD*) and -Areal specific energy absorption of the gradient cell core are reduced by 13% and 1.9%, respectively, compared with the uniform cell core. Li et al.^50^ performed blast tests on metallic sandwich structures with density-variant aluminum honeycomb cores. The results indicated that panels featuring a decreasing relative density gradient exhibited enhanced blast mitigation capabilities relative to their uniform-density counterparts.

While these advancements represent significant progress in enhancing the blast resistance of auxetic honeycomb cores, further improvements are still necessary to meet the demanding real-world protection requirements. Despite the various structural and material modifications, the explosion-proof performance of sandwich panels incorporating auxetic honeycomb cores remains insufficient in many applications, especially in high-intensity blast scenarios. To bridge this gap, the present study aims to develop a novel blast-resistant sandwich structure based on a variable stiffness auxetic honeycomb core. This paper mainly studies the explosion response of honeycomb sandwich structures with variable stiffness. A finite element model of the composite core was established to simulate the deformation patterns and energy absorption behavior. The paper systematically investigates the influence of different design strategies, including relative core densities, geometric parameters, face sheet thickness, stand-off distance, and gradient configurations, on the deformation mechanisms and over all the anti-explosion performance of the target structure.

## Problem description

### Geometry description

**Fig. 1:**
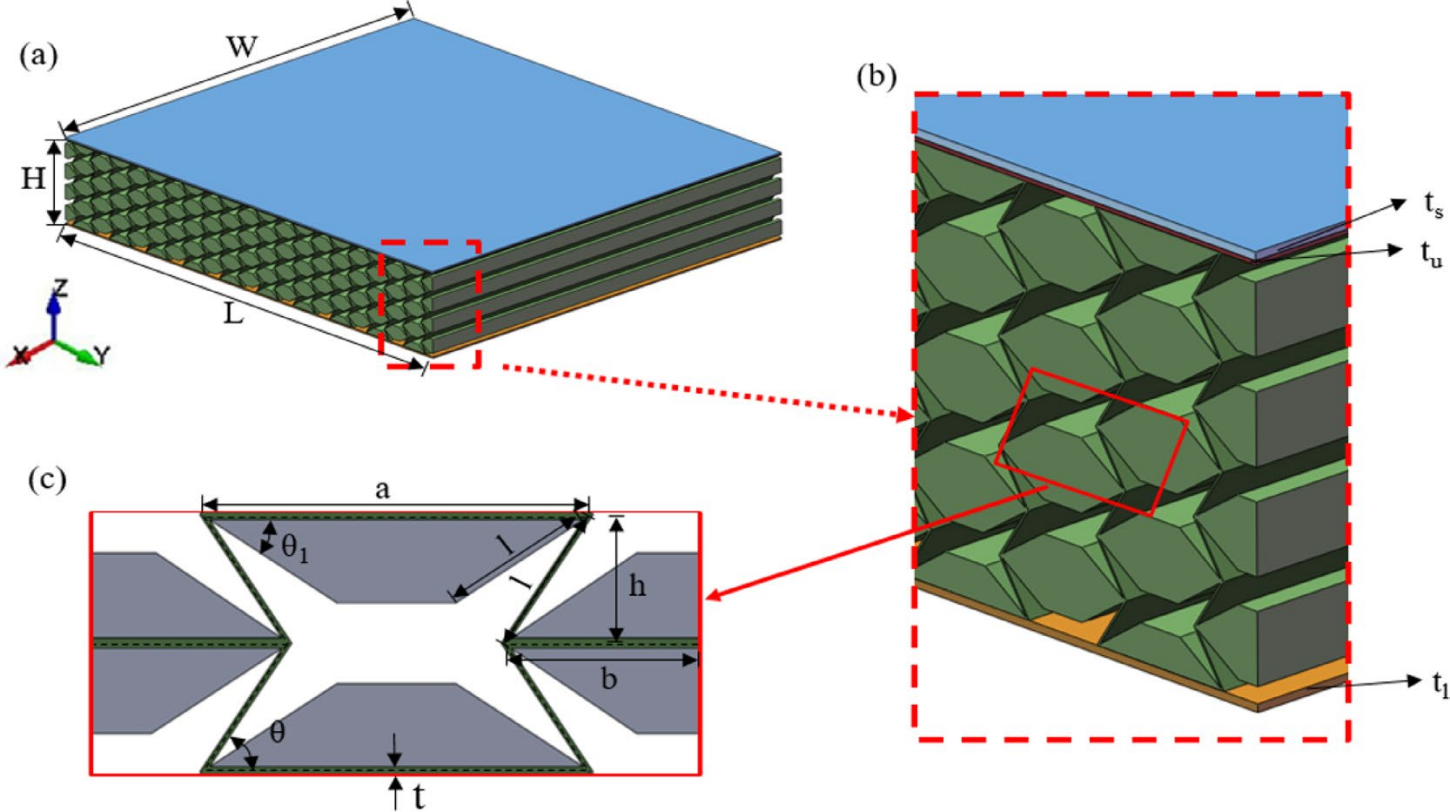
(**a**) Schematic of VSRE auxetic core sandwich panel; (**b**) Enlarged view; (**c**) VSRE cellular configuration. The sandwich panel consists of a steel plate, upper and lower panels, and a VSRE honeycomb core with total dimensions in the *X*, *Y*, and *Z* directions of *L*×*W*×*H*.

The variable stiffness re-entrant (VSRE) core sandwich panels are composed of a steel plate, upper and lower panels, and an auxetic re-entrant honeycomb core. The VSRE sandwich structure exhibits dimensional parameters *L*×*H*×*W* along the respective X-, Y-, and Z-axes, as illustrated in Fig. 1a, the variable stiffness honeycomb core has repetitive cell numbers of 10 and 4 in the *X* and *Z* directions^37,51^. Specifically, the panel’s length is *L* = 500 mm, width *W* = 550 mm, and height *H* = 101 mm. Figure 1b illustrates the thickness of the steel plate (*t*s = 1 mm), upper panel (*t*u = 1 mm), and lower panel (*t*l = 1 mm). Additionally, Fig. 1c displays the outline of the honeycomb core, where the re-entrant unit cell is divided into an initial region (green) and a variable stiffness region (gray). The parameter values for the variable stiffness region are as follows: length *a* = 35 mm, length *b* = 20 mm, height 2*h* = 24 mm, wall thickness *t* = 0.5 mm, and re-entrant angle *θ* = 58°. The Variable Stiffness Factor (VSF) is a geometric design parameter used to quantitatively control the densification strain of re-entrant structure. It is defined as the ratio of the adjustment angle of the variable stiffness region (*θ*_1_) to the re-entrant angle (*θ*). By changing the VSF value, the contact sequence of the internal ribs during the compression process of the structure can be controlled, thereby actively regulating the stiffness and energy absorption characteristics. To easily and accurately adjust the densification strain, only the variable stiffness angle *θ*1 is variable. In this study, we only consider the case 2*l* < *a* to avoid local buckling caused by misalignment of the inner corners during compaction and to ensure effective contact and densification within the predicted region of variable stiffness.

1 $$\eta =1 - \frac{{{\theta _1}}}{\theta }$$

In this context, *η* represents the proportion of the design (*η* represents VSF), *θ*_1_ represents the variable stiffness angle, and *θ* represents the re-entrant angle. the range of values for the VSF of the inner auxetic cell can be expressed as follows:

When *a* > 2*l*, we can estimate the values of *θ*_1_ and *η* using the following equations:

2 $$0 \leqslant {\theta _1} \leqslant \theta, \quad 0 \leqslant \eta \leqslant 1$$

The relative density of a variable stiffness re-entrant honeycomb is defined as the ratio of the cell wall volume to the total dimensions *L*×*H*×*W*, as illustrated in Fig. 1. It can be expressed by Eq. (3) in terms of the cell parameters.

3 $${\rho _r}=\frac{{(2l+a)t+({a_1} - \cos {\theta _1}l)l\sin {\theta _1}}}{{la\sin \theta }}$$

**Fig. 2:**
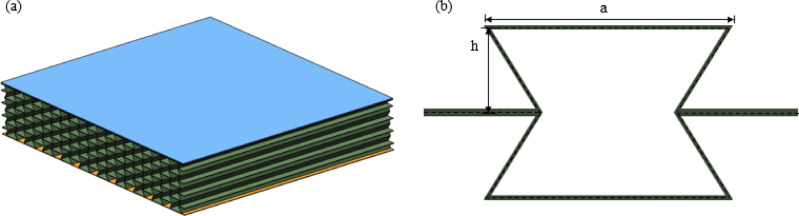
(**a**) Schematic of RE-core sandwich panel; (**b**) RE cell elements. the RE-core core panel has the same macroscopic dimensions, sandwich layer dimensions, upper and lower panel thicknesses, and number of cells in both orthogonal directions as the VSRE core core panel.

In order to objectively evaluate the enhanced explosion-proof performance of the variable stiffness re-entrant (VSRE) sandwich panel compared with the traditional re-entrant (RE) structure, we constructed a model with the same mass (RE_*m*) and equivalent wall thickness (RE_*t*), as shown in Fig. 2. To conduct a fair performance comparison, all material parameters and geometric parameters, including the overall panel size, cell specification, panel thickness and cell repeat count, are consistent with VSRE. It was finally determined that the wall thickness of the RE_*m* core was 3.6 mm.

### Finite element modeling

**Fig. 3:**
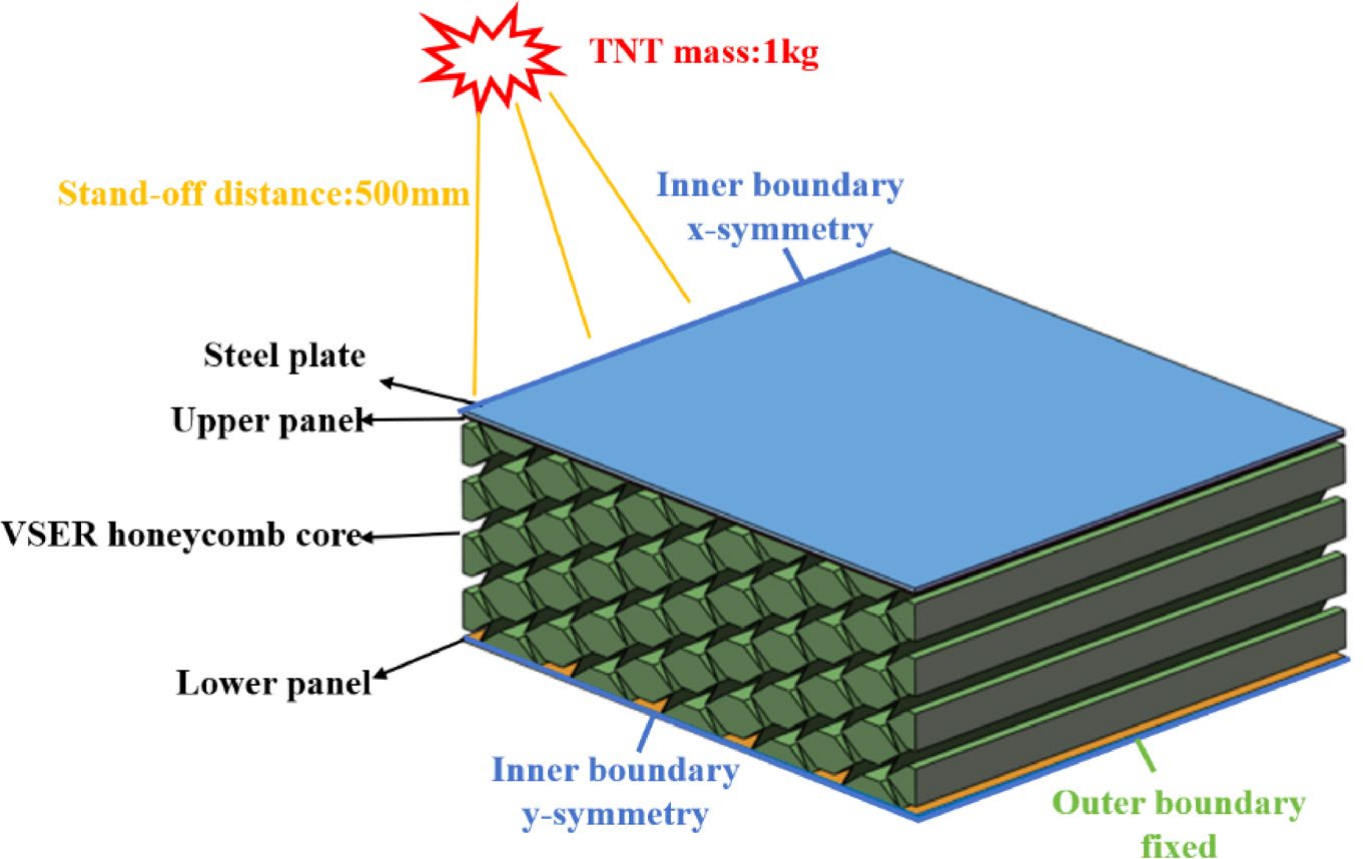
Finite element model of VSRE core sandwich panel. Due to the symmetric structure, only a quarter of the sandwich panel is modeled to reduce the computation time.

To conduct the finite element modeling of the blast response in the RE core sandwich panel, we utilized the commercial software Solidworks^®^ and selected the nonlinear explicit solver LS-DYNA. Figure 3 illustrates that, due to the simulation’s symmetry, we adopt the quarter-symmetric modeling method to optimize computing resources while maintaining simulation accuracy. The VSRE model, depicted in Fig. 1c, was employed for the cell, with all cell walls represented by solid164 element. For the sake of comparison, the same simulation setup was implemented for the RE core sandwich panels. The convergence analysis of the grid density determined an optimal element size of 2 mm, resulting in a total of 154,462 elements in the model.

**Fig. 4:**
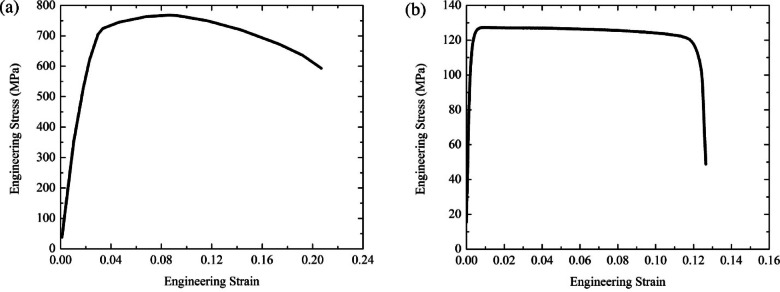
Stress–strain curves of (**a**) Grade 800 HSS and (**b**) aluminum alloy AA6061^37^.

In the study conducted by Qi^37,51^, according to the relevant regulations, 800 grade High Strength Steel (HSS) plate and AA6061 aluminum alloy were used to manufacture steel plate and sandwich plate (covering the initial region in Fig. 1.c and the variable stiffness region) respectively. To align with these experiments, this paper also employed aluminum alloy as the core material. For the simulation, intrinsic models of 800 HSS and AA6061 were established in LS-DYNA using MAT_PIECEWISE_LINEAR_PLASTICITY, allowing definition of their stress-strain curves. Table 1 presents a summary of the input parameters for HSS and AA6061 in LS-DYNA. The stress-strain curve is shown in Fig. 4.

**Table 1:** Input parameters of HSS and AA6061 in FE models^37^.

Material	Part	Density/(kg/m^3^)	E/GPa	PR	SIGY/MPa	FAIL	C/s^− 1^	*P*
G800HSS	Steel plate	7850	223	0.3	789	0.2	3200	5
AA6061	Sandwich panel	2710	40.3	0.33	113	0.12	–	–

Notes: G800HSS—High-strength steel; AA6061—Aluminum alloy; E—Elastic modulus; PR—Poisson’s ratio; SIGY—Yield stress; FAIL—Failure strain; C—Strain rate ratio coefficient; P—Define the elastoplastic model of the material.

The sandwich panel bears the explosive load, and the explosive position of the charge is directly above the steel plate. A simulated blast load, using the CONWEP formula, represented an air blast explosion^52^. This device uses 1 kg of tnt equivalent explosives and is placed at a distance of 500 millimeters from the center of mass of the base plate. The finite element model’s boundary conditions were twofold: the inner boundary applied the symmetry condition, while the outer boundary implemented the fixed condition, as illustrated in Fig. 3. The symmetry boundary conditions restricted translational degrees of freedom along their respective normal directions for nodal points located on both XOZ and YOZ symmetry planes. The completely fixed boundary conditions eliminate all six degrees of freedom at the constraint nodes to prevent zero-displacement penetration. Specifically, the AUTOMATIC_SURFACE_TO_SURFACE contact algorithm was utilized for the nodes between the steel and the upper panel, and between the honeycomb core and the upper and lower panels. Furthermore, AUTOMATIC_SINGLE_SURFACE was used for all components to prevent self-penetration. The static friction coefficient of all contact interfaces is 0.3 and the dynamic friction coefficient is 0.2.

## Validation of the FE model

**Fig. 5:**
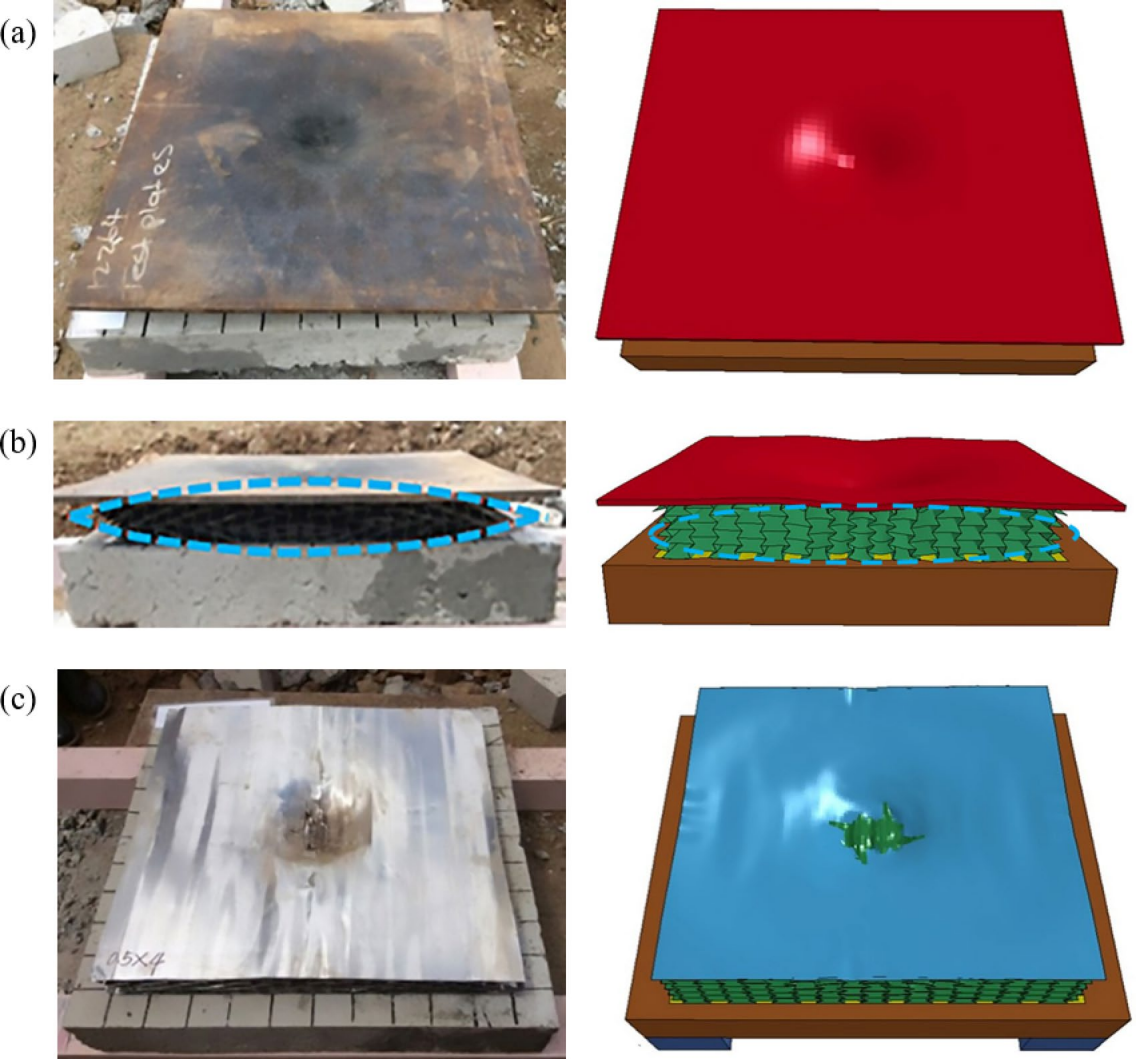
RE sandwich panel test and simulated damage. (**a**) Top view of the sandwich panel, (**b**) Side view of the sandwich panel, (**c**) Top view of RE sandwich panel^37^.

The explosion response of an auxetic honeycomb core sandwich panel (RE) was investigated using experimental tests and numerical simulations, employing the same finite element modeling approach. The RE sandwich structure was positioned atop a reinforced concrete substrate, with a steel cladding layer subsequently installed over the assembly. A cylindrical charge was centrally aligned over the steel cladding to induce blast loading. Figure 5 provides a comparative analysis of the RE sandwich composite’s deformation patterns from experimental and numerical simulations. In the top view of Fig. 5a, it is observed that the explosion caused a depression and localized plastic deformation in the upper layer of the steel plate, but no fracture occurred. The honeycomb core layer of the RE sandwich panel experienced some degree of fracture, and the simulated damage shape closely matched the test results. Figure 5b presents a cross-section comparison, which reveals a more pronounced deformation in the cellular core layer of the RE sandwich panel, indicating material densification towards the middle of the panel. For comprehensive investigation of the strain evolution in the intermediate composite structure subjected to blast loading, the upper steel lamina was detached (Fig. 5c), enabling direct observation of internal deformation mechanisms. Partial fracture initiation manifested within the RE core material, while the numerically predicted damage morphology exhibited strong congruence with experimental outcomes. The depression deformation of the steel plate contributed to a larger deflection of the blast shock wave, making the RE sandwich panel exhibit greater plasticity. Furthermore, the lower reinforced concrete substrate exhibited negligible structural compromise in both experimental tests and numerical simulations. The close agreement between observed and predicted deformation patterns confirms the accuracy of the modeling approach in capturing the structural response under blast loading conditions.

**Fig. 6:**
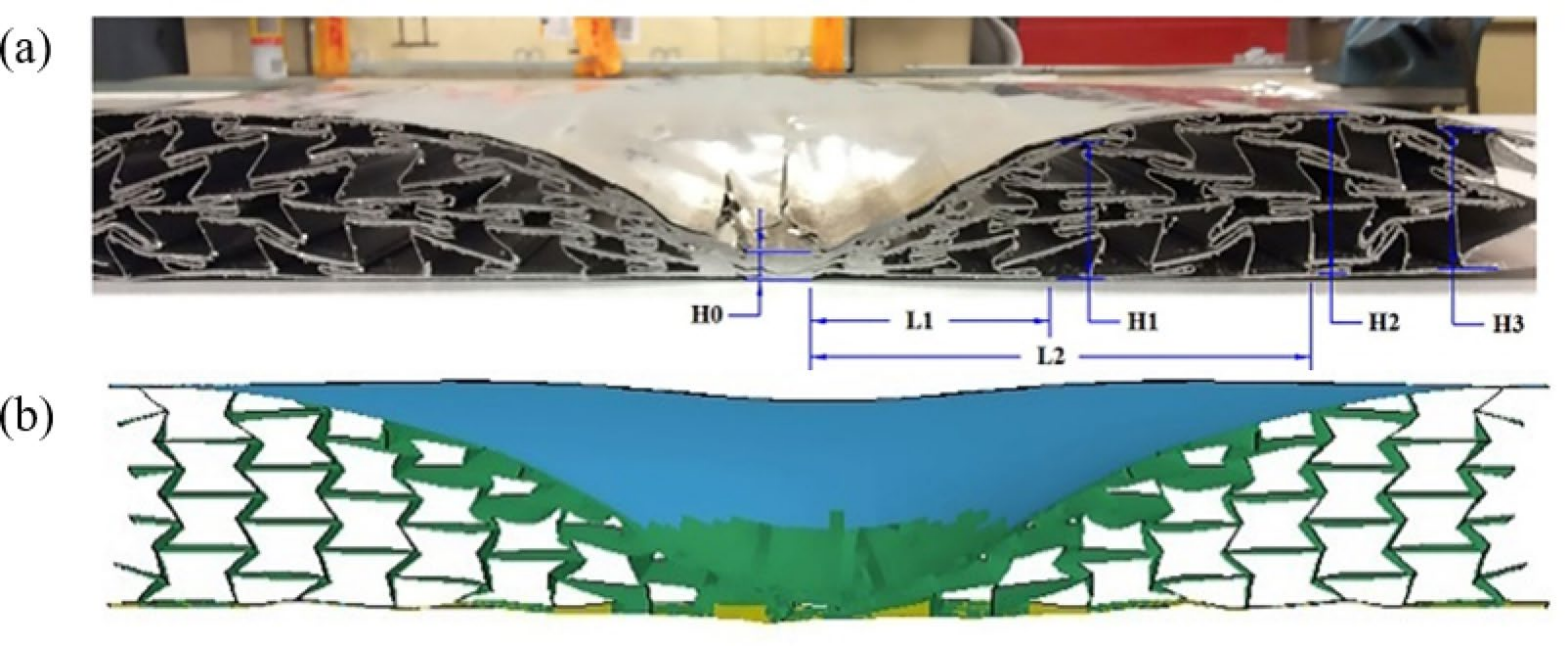
(**a**) Intermediate sections of sandwich panel specimens, (**b**) corresponding finite element predictions after field blast tests^37^. *H*_0_ is the height at the center point, *H*_2_ is the maximum height, *L*_2_ is the transverse distance from the center to the highest point, *H*_1_ is the height between the center midpoint and the highest point, *L*_1_ is the transverse distance from the center to the intermediate point, and *H*_3_ is the height at the edge.

Figure 6 illustrates the deformation of the middle section of the sandwich panel specimen and the corresponding finite element prediction results after the blast test. To rigorously verify the finite element modeling methodology, the simulated strain metrics illustrated in Fig. 6 were benchmarked against empirical measurements (Table 2) through a quantitative validation framework. The comparison revealed that the numerical predictions closely matched the experimental results with regards to the intermediate cross-section and deformation parameters. The high congruence between computational simulations and empirical measurements substantiates the robustness of the FE modeling methodology implemented in this investigation.

**Table 2:** Comparison of intermediate cross-section deformation data obtained from field blasting tests and numerical simulations^37^.

Parameter	H_0_ /mm	H_1_ /mm	H_2_ /mm	H_3_ /mm	L_1_ /mm	L_2_ /mm
Experimental measurement	10.0	45.8	50.0	46.4	91.4	182.8
Numerical prediction	10.89	49.92	48.6	49.2	94.2	190.4
Relative error	1.9%	9.0%	2.8%	6.0%	3.1%	4.0%

## Results and discussion

This section begins by comparing the blast resistance of the VSRE sandwich panels with the RE sandwich panels to emphasize the advantages of the VSRE sandwich panels. The subsequent analysis scrutinizes the strain mechanisms, ultimate displacement magnitudes, and energy attenuation characteristics inherent to the dual variants of sandwich panels, thereby elucidating the fundamental principles that govern their mechanical response and structural resilience. The blast mitigation performance of VSRE cored sandwich panels is quantitatively assessed with respect to five key variables: re-entrant angles (*θ*), VSF magnitude, stand-off spacing, thickness *t*, and gradient parameters.

### Dynamic response of sandwich panel

**Fig. 7:**
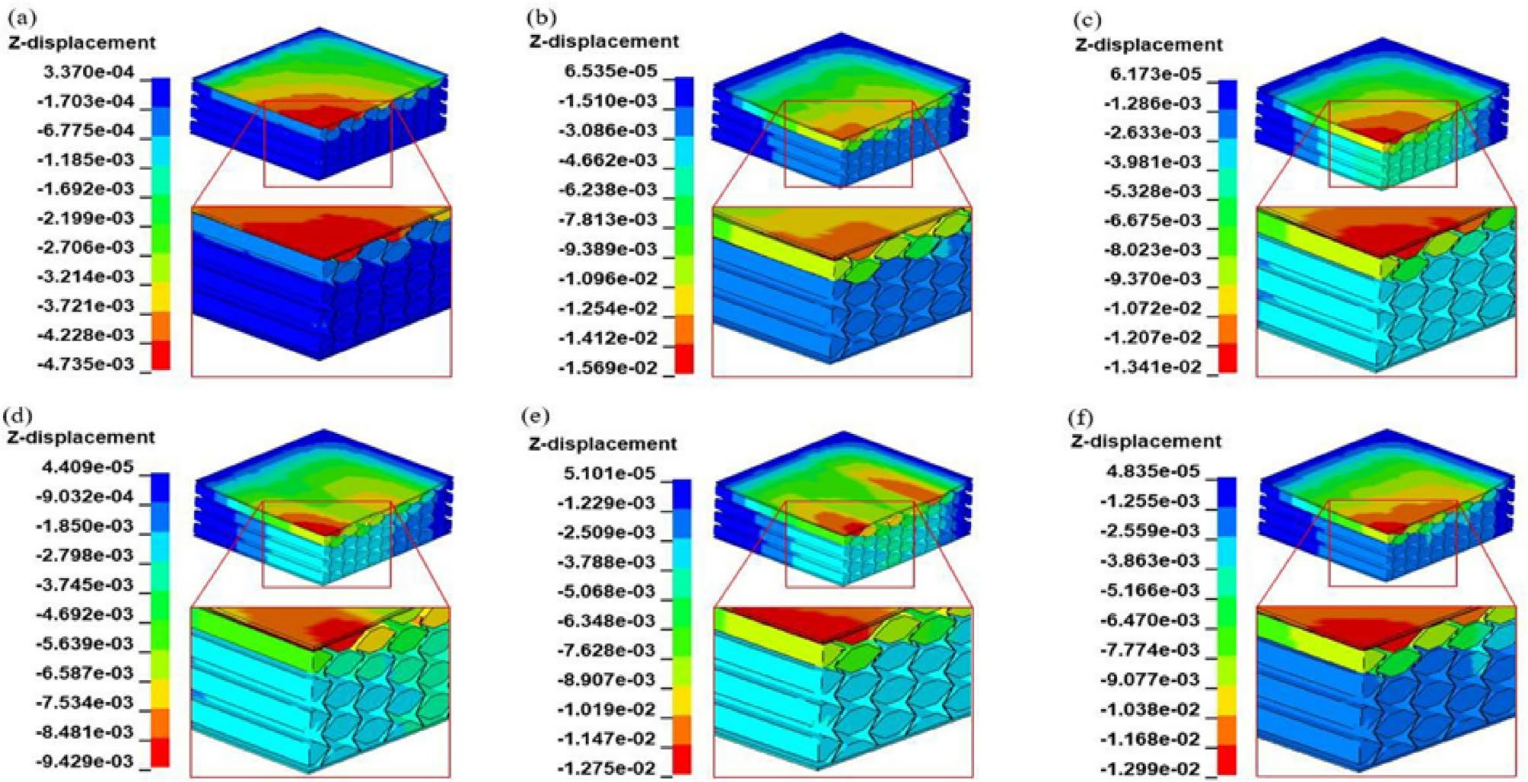
Typical deformation patterns of VSRE sandwich panels. (**a**) *t* = 40 µs, (**b**) *t* = 100 µs, (**c**) *t* = 220 µs, (**d**) *t* = 360 µs, (**e**) *t* = 440 µs, (**f**) *t* = 930 µs.

In the modeling explosion loading section, the simulation in Fig. 7 illustrates the deformation process of the VSRE structure. The overall deformation reveals a dome shape starting from the center due to the negative Poisson’s ratio effect. Consequently, the VSRE core sandwich panel concentrates at the center loading place, leading to increased structural stiffness and a more homogeneous distribution of the explosion energy. The deformation process can be roughly categorized into three phases including the loading phase, the compression phase, and the rebound phase. Initially, the blast load results in zero displacement at 0 ms, accelerating the front plate. Subsequently, the front panel gradually compresses the core structure, leading to increased displacement until the largest deflection occurs at the center point of the lower panel (40–100 µs). Finally, during the rebound phase (100–930 µs), the sandwich panel exhibits low-amplitude vibrations before gradually stabilizing into a static state. Similar post-blast dynamic responses have also been reported by Wang et al.^48^, who observed comparable behavior in graded foam-core sandwich panels subjected to blast loading.

**Fig. 8:**
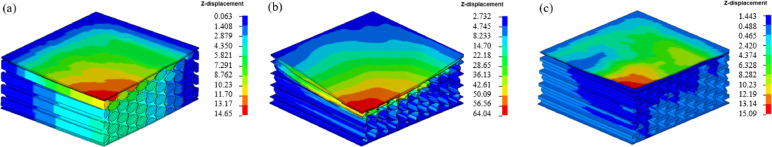
FE predicted deformed shapes of sandwich panels with different cores of (**a**) VSRE; (**b**) RE_*t*; and (**c**) RE_*m*.

To elucidate the strain evolution mechanics, Fig. 8 presents the distortion patterns of three auxetic-cored sandwich structures under peak vertical deflection (*maxD*). In the case of the RE_*t* core, lacking a variable stiffness area and prone to deform under compression conditions, the core folds against the inclined wall, leading to an overall unstable deformation. Furthermore, the deformation is most significant along the thickness direction of the core panel (Fig. 8a). Conversely, the RE_*m* core exhibits a non-uniform deformation due to the larger gap between folded walls resulting from a thicker cell wall compared to the VSRE core. In contrast, the VSRE core demonstrates a more stable overall deformation as the ribs initially come into contact with the variable stiffness area. The stabilized state improves energy dissipation efficiency and strengthens the core architecture, thereby achieving negligible structural distortion.

**Fig. 9:**
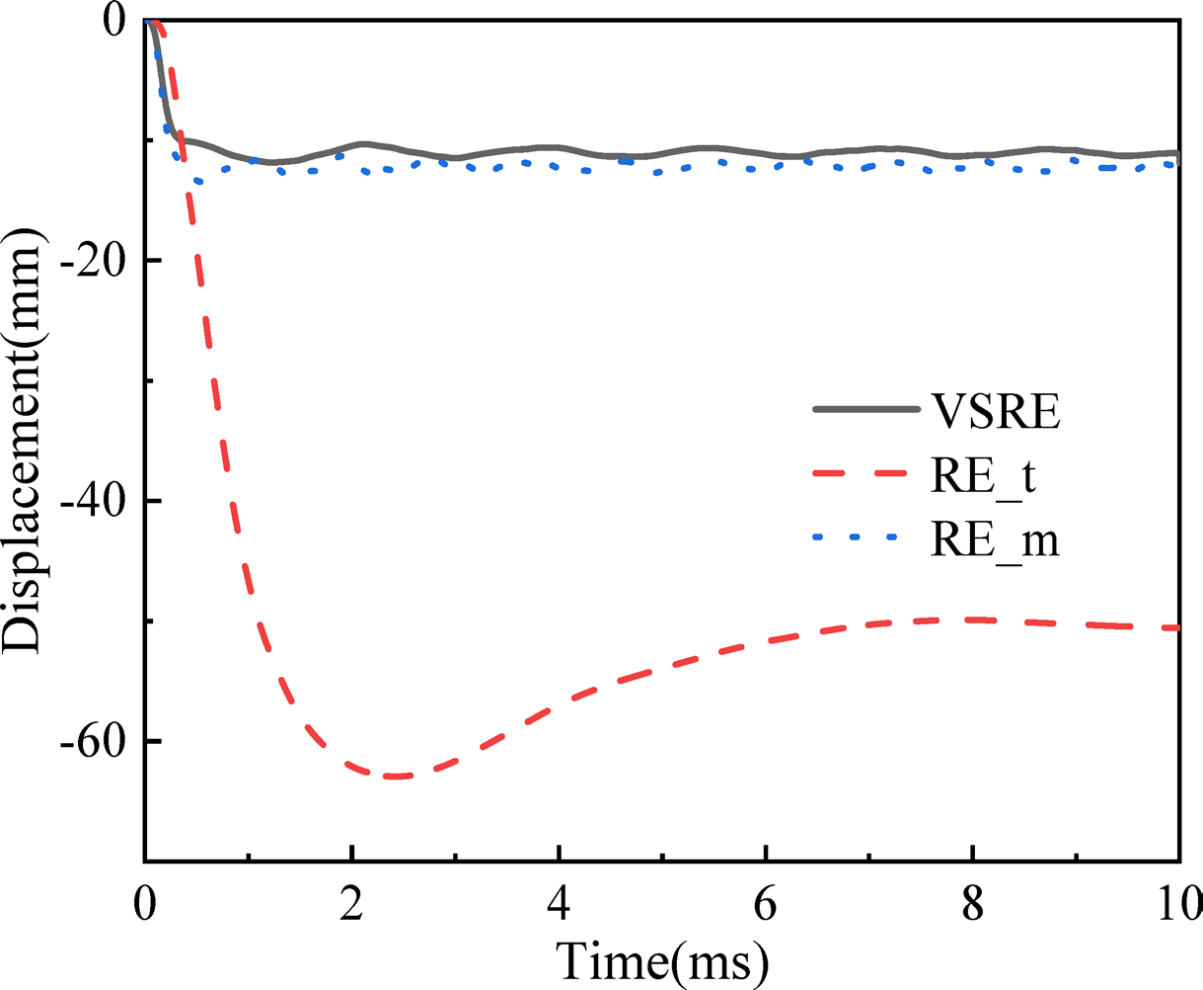
*Z*-displacement histories of the midpoints of different sandwich lower panels. The black solid line, blue dashed line, and red dotted line indicate the VSRE-based core, RE core of the same quality, and RE core of the same cell wall thickness, respectively.

To elucidate the anti-explosive behavior of the composite sandwich architecture, computational quantification was performed to characterize its dynamic mechanical response under blast loading. Figure 9; Table 3 quantitatively characterize the temporal displacement evolution at the central region of the bottom plate for three distinct core configurations: VSRE, RE_*t*, and RE_*m* cores. It is observed that the time taken to reach the *maxD* of the lower panel differs for each core, 1.19 ms for the VSRE core, 3.29 ms for the RE_*m* core, and 2.49 ms for the RE_*t* core. Furthermore, the *maxD* values of the lower panel are 11.86 mm, 13.54 mm, and 62.95 mm for the VSRE, RE_*m*, and RE_*t* cores, respectively. Table 3 presents bending displacement and energy dissipation capacity of the sandwich panel across three distinct core configurations. With a 14.2% higher *maxD* observed in the RE*_m* core relative to the VSRE configuration, this comparison conclusively demonstrates the superior blast mitigation capability of VSRE cored sandwich panels.

**Table 3:** Maximum Deflection and energy absorption of sandwich panels with different cores.

Series	Honey-comb cores	VSF	Stand-off distance(mm)	Re-entrant angle (°)	t_u_ (mm)	t_l_ (mm)	maxD (mm)	EA_s_ (kJ)	EA_u_ (kJ)	EA_h_ (kJ)	EA_l_ (kJ)	EA (kJ)
1	VSRE	40	500	58	1	1	11.86	0.76	0.36	3.45	0.04	4.61
	RE_*m*	40	500	58	1	1	13.54	0.17	0.48	3.29	0.09	4.03
	RE_*t*	40	500	58	1	1	62.95	2.39	0.26	0.93	0.05	3.63
2	VSRE	40	500	58	1	1	11.86	0.76	0.36	3.45	0.04	4.61
	VSRE	60	500	58	1	1	22.21	1.01	0.37	2.65	0.04	4.07
	VSRE	80	500	58	1	1	35.83	1.35	0.35	2	0.04	3.74
	VSRE	100	500	58	1	1	62.95	2.39	0.26	0.93	0.05	3.63
3	VSRE	40	500	58	1	1	11.86	0.76	0.36	3.45	0.04	4.61
	VSRE	40	1200	58	1	1	4.81	0.21	0.26	1.58	0.03	2.08
	VSRE	40	2000	58	1	1	1.31	0.03	0.05	0.33	0.005	0.415
4	VSRE	40	500	45	1	1	9.86	1.35	0.38	1.05	0.01	2.79
	VSRE	40	500	50	1	1	10.30	1.17	0.41	1.42	0.06	3.06
	VSRE	40	500	55	1	1	10.95	1.03	0.34	1.93	0.1	3.4
	VSRE	40	500	60	1	1	11.99	0.76	0.36	2.65	0.37	4.14
5	VSRE	40	500	58	1	1	11.86	0.76	0.36	3.45	0.04	4.61
	VSRE	40	500	58	1.5	1	10.94	0.79	0.36	2.81	0.03	3.99
	VSRE	40	500	58	2	1	10.3	0.82	0.37	2.36	0.03	3.58
	VSRE	40	500	58	2.5	1	9.81	0.85	0.37	2.14	0.02	3.38
	VSRE	40	500	58	3	1	9.6	0.88	0.38	2.01	0.01	3.28
6	VSRE	40	500	58	1	1.5	10.32	0.75	0.34	3.43	0.04	4.56
	VSRE	40	500	58	1	2	9.22	0.72	0.31	3.41	0.03	4.47
	VSRE	40	500	58	1	2.5	8.36	0.69	0.29	3.37	0.03	4.38
	VSRE	40	500	58	1	3	8.01	0.66	0.26	3.35	0.02	4.29
7	VSRE	G1	500	58	1	1	4.09	0.91	0.37	4.21	0.02	5.51
	VSRE	G2	500	58	1	1	4.22	0.70	0.24	3.18	0.03	4.15
	VSRE	G3	500	58	1	1	2.04	0.91	1.01	4.95	0.02	6.89
	VSRE	G4	500	58	1	1	2.17	0.25	0.32	3.74	0.06	4.37

Note: *t*u, *t*l represent the thickness of the upper panel and the thickness of the lower panel respectively, and *EA*s, *EA*u, *EA*h, *EA*l represent the energy absorption values of the steel plate, the upper panel, the honeycomb and the lower panel respectively.

**Fig. 10:**
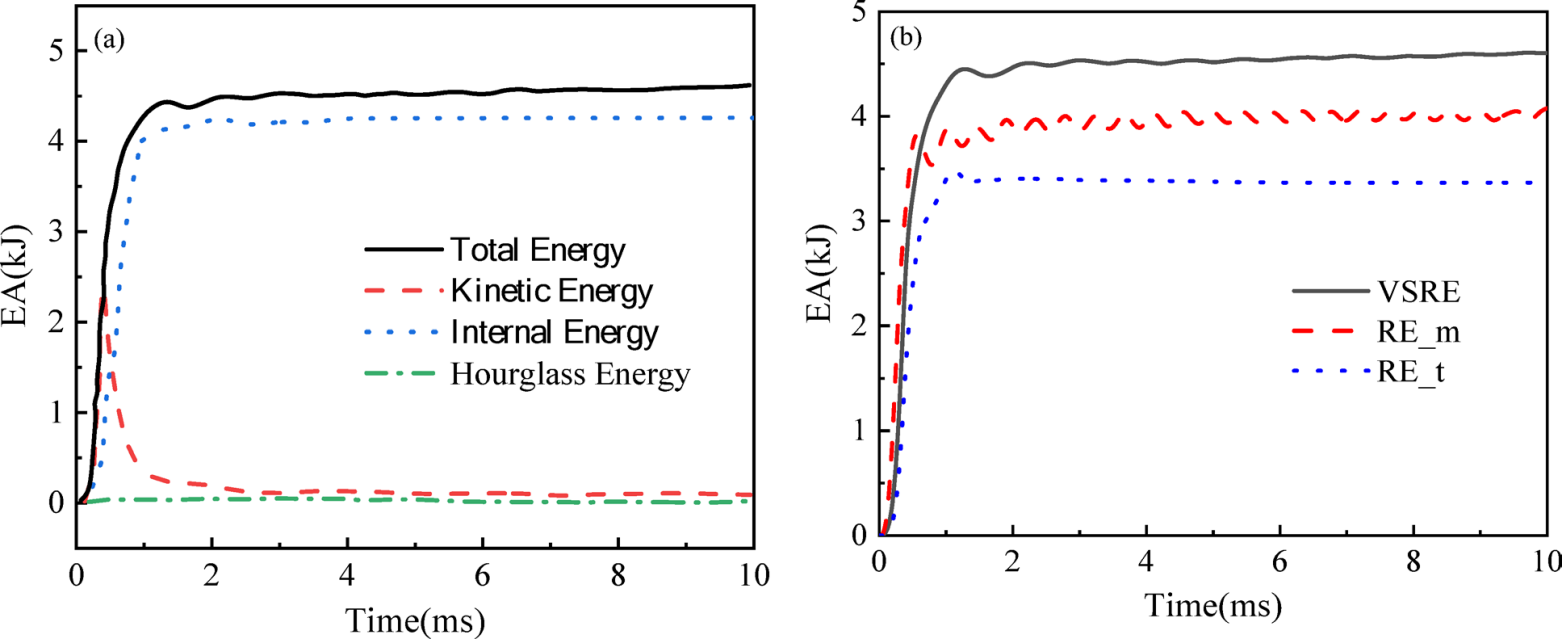
(**a**) Energy history of VSRE structures; (**b**) Energy history of *EA* sandwich plates with different cores. The black solid line, the red dashed line, and the blue dotted line indicate the VSRE core-based sandwich plate, the RE core sandwich plate with the same mass, and the RE core sandwich plate with the same cell wall thickness, respectively.

Figure 10a displays the energy-time curve of the VSRE core sandwich panel. Experimental findings confirm that the combined contributions of kinetic, internal, and hourglass energies maintain strict equivalence with the system’s overall energy throughout all temporal stages. Additionally, the ratio of hourglass energy to total energy remains below the allowable limit, further confirming the accuracy of the simulation results. Consequently, energy equilibrium is consistently maintained across all numerical models developed in this work, which rigorously corroborates the credibility of the computational outcomes. In Fig. 10b, the energy-time curves for different core sandwich panels are presented. The absorbed energy initially increases and then stabilizes at the final energy, with absorption values of 4.61 kJ, 4.08 kJ, and 3.63 kJ for the sandwich panel with VSRE, RE_*m*, and RE_*t* cores, respectively. Compared to the RE_*m* and RE_*t* cores, the VSRE core exhibits a 12.9% and 27.5% improvement in *EA*, respectively. Table 3 delineates energy dissipation patterns across sandwich panels with varying core designs. Analysis reveals that the honeycomb structure and rear panel account for over 85% of total energy dissipation. As displayed in Table 3, the energy absorption percentage of the honeycomb core is 74.8%, 81.6%, and 25.6% for the VSRE, RE_*m*, and RE_*t* cores, respectively, while the energy absorption percentage of the lower sheet is 0.8%, 2.2%, and 1.4% for the respective cores. Thus, the VSRE configuration demonstrates dual advantages: it amplifies the composite’s overall energy dissipation capability while significantly boosting the auxetic core’s proportional contribution to total energy mitigation, outperforming both RE_*m* and RE_*t* designs.

In summary, VSRE cores exhibit less displacement and higher energy absorption compared to RE cores of identical mass and wall thickness. The observed behavior stems from the intrinsic strain evolution processes governing the VSRE core’s structural response.

### Effect of different VSF value

**Fig. 11:**
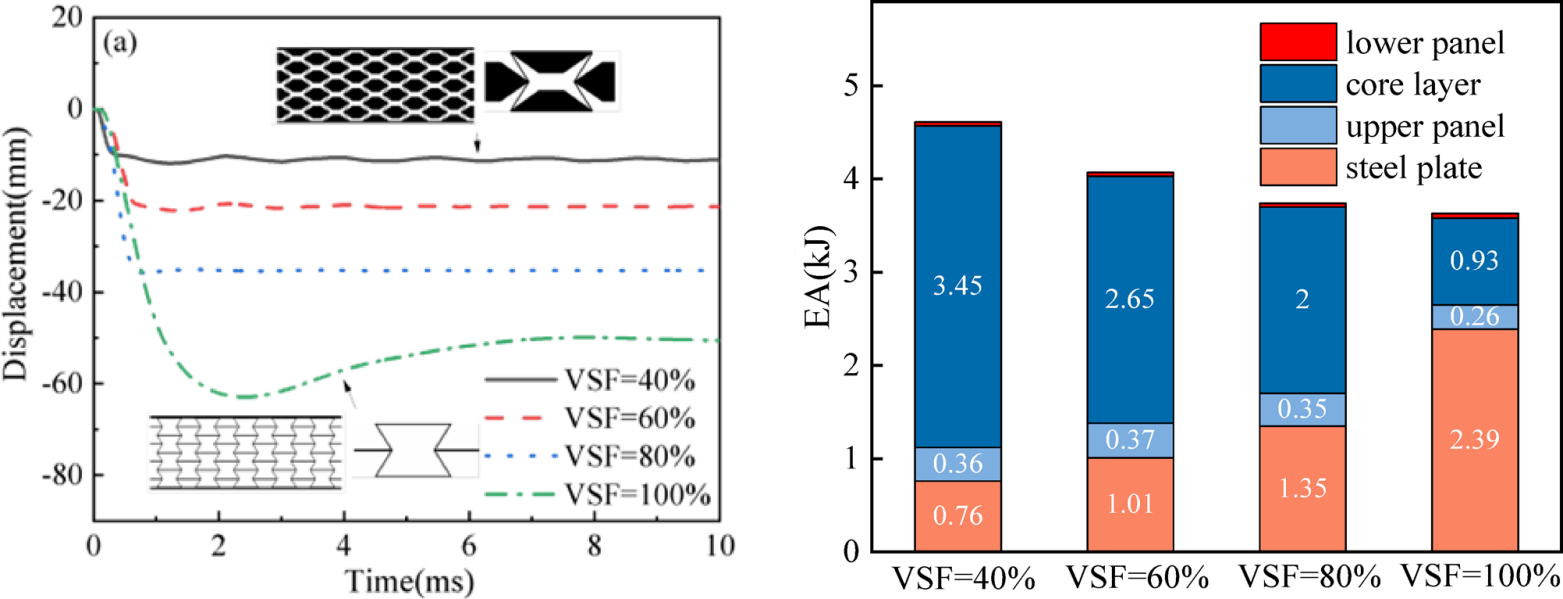
(**a**) *maxD* of sandwich panels with different VSFs; (**b**) *EA*_i_ distribution between sandwich panels with different VSFs.

For enhanced verification of the four variable stiffness models configured at VSF gradations of 40%, 60%, 80%, and 100%, corresponding computational frameworks were established. Figure 2 illustrates the geometric configurations of the characteristic volumetric domains. Following this, computational structural analyses were systematically conducted on all model variants. The displacement time histories of the four models with VSF values of 40%, 60%, 80%, and 100% are presented in Fig. 11a. The variation in VSF leads to a change in the sandwich panel’s topology, as illustrated in Fig. 1c for a VSF of 40%. As VSF decreases, both *maxD* and *EA* show improvement. Specifically, the *maxD* and *EA* measure 11.86 mm and 4.61 kJ for VSF = 40%, while they are 62.95 mm and 3.63 kJ for VSF = 100%. Adjusting the VSF allows for a 430.1% reduction in the *maxD* of a sandwich panel with VSF = 40% compared to VSF = 100%, as well as a 26.9% increase in the *EA* of a sandwich panel with VSF = 40% compared to VSF = 100%. On the one hand, a larger VSF implies fewer cells in a fixed cross-sectional area and a lower overall stiffness of the sandwich plate, which results in a larger *maxD*.

Figure 11b and Table 3 illustrate the distribution of *EA* among sandwich plates with different VSF values. As VSF increases, EA decreases, resulting in a reduction in the ratio of *EA*c to *EA*, while the ratio of *EA*_l_ to *EA* becomes larger. For instance, with VSF = 60%, the ratios of *EA*_c_ and *EA*_l_ to *EA* are 65.1% and 0.98%, respectively. Similarly, for VSF = 80%, the ratios of *EA*_c_ and *EA*_l_ to *EA* are 53.5% and 1.16%, respectively. The VSF alters the structural deformation mechanism and exerts a significant influence on *EA*. A lower VSF value enhances *EA* by promoting early cell densification and stronger reverse deformation properties, thereby improving stress redistribution and reducing overall deformation. Therefore, adjusting VSF can optimize the trade-off between stiffness and energy absorption.

### Effect of different re-entrant angles

**Fig. 12:**
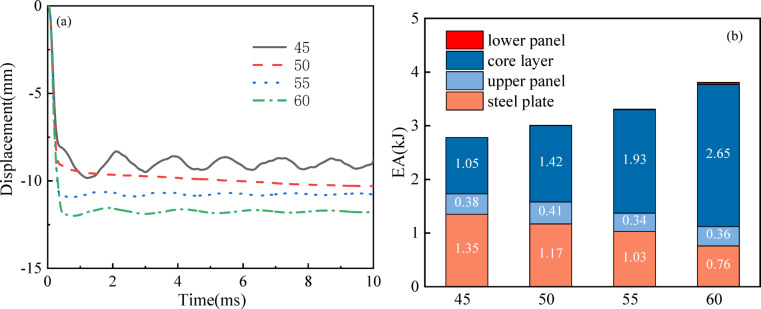
(**a**) *maxD* for sandwich plates with different re-entry angles *θ*; (**b**) *EA*_i_ distribution between sandwich plates with different *θ*.

At a VSF of 40%, four parametric models spanning 45°–60° re-entrant angles (*θ*) were constructed to analyze the influence of *θ* on structural deformation mechanisms. Progressive elevation of *θ* correlates with amplified panel deflection (*maxD*), which enhances energy dissipation and material utilization density, ultimately elevating the structural system’s global load resistance. Figure 12a depicts the *maxD* and *EA* of sandwich panels at different re-entrant angles, showing an increase in both *maxD* and *EA* as *θ* increases from 45° to 60°. The total height of the protection system is found to increase with *θ*, leading to a reduced distance between the blast and the steel plate due to the fixed stand-off distances. Consequently, the load applied to the steel cover increases, resulting in an increased *EA*. The increased total height also allows for more room for deformation of the protection system, leading to a subsequent increase in *maxD*.

The impact of *θ* on *EA* distribution is demonstrated in Fig. 12b. With the increase in *θ*, the ratio of *EA*c to *EA* becomes smaller, while the ratio of *EA*l to *EA* becomes larger. Specifically, when *θ* = 45°, the ratios of *EA*c and *EA*l to *EA* are 37.63% and 0.36%, respectively, and when *θ* = 60°, the ratios are 64.01% and 8.94%, respectively. For instance, there is a significant increase in *maxD* from 9.86 to 11.65 mm, representing an 18.15% increase, while *EA* increases from 2.79 to 4.14 kJ, an increase of 48.38%. Elevated *θ* values induce diminished flexural strain within cellular walls, concurrently lowering the structural rigidity of the composite panel.

The re-entrant angle *θ* significantly governs the failure mechanism and energy absorption characteristics of the structure. Numerical analysis demonstrates that structures with smaller *θ* values (45°) predominantly exhibit localized folding deformation at the re-entrant joints, creating concentrated stress regions that accelerate core densification. In contrast, configurations with larger *θ* angles (60°) develop a bending-dominated deformation mode characterized by distributed plastic hinge formation, which enhances structural stability and blast resistance. This mechanistic transition explains the performance trade-off observed between energy absorption (*EA*) and maximum displacement (*maxD*), where increasing *θ* from 45° to 60° improves *EA* by 48.38% while increasing *maxD* by 18.15%. These findings highlight the critical importance of optimizing the re-entrant angle to achieve an optimal balance between energy dissipation capacity and displacement control in blast-resistant sandwich composite design.

### Effect of different stand-off distance

**Fig. 13:**
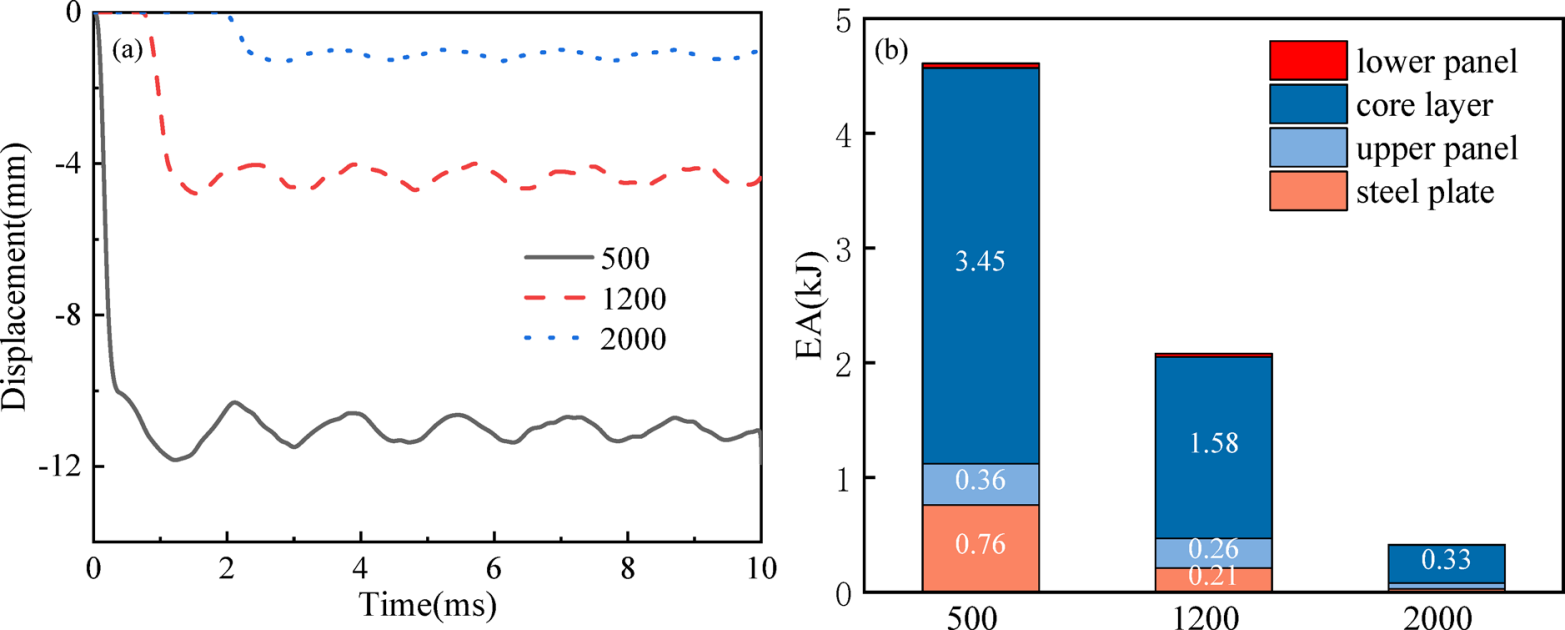
(**a**) *maxD* of sandwich panels at different detonation distances; (**b**) *EA*i distribution between sandwich panels at different stand-off distances.

Simulations are conducted in this section using honeycomb cores with VSF = 40% and 500 g of TNT explosive detonated at three different distances from the center of the building, including 500 mm, 1200 mm, and 2000 mm. Figure 13a delineates the temporal displacement evolution at the central region of the front panel under varying stand-off distances (*SOD*). As the *SOD* escalates from 500 to 1200 mm, the back sheet’s maximum displacement diminishes progressively from 11.86 mm to 4.81 mm, representing a 7.05 mm reduction in peak deflection. Similarly, increasing the *SOD* from 1200 to 2000 mm results in a decrease in peak deflection from 4.81 to 1.31 mm, a decrease of 3.5 mm. In essence, the magnitude of the increase in deflection is significantly greater in the former case (*SOD* from 500 mm to 1200 mm with an increase of 7.05 mm) compared to the latter case (*SOD* from 1200 to 2000 mm with an increase of 3.5 mm). The observed discrepancy stems from the exponentially decaying shock wave pressure profile as *SOD* escalates under blast loading conditions.

Figure 13b and Table 3 illustrate the relationship between *SOD* and energy absorption (*EA*) in VSRE core sandwich panels. As the *SOD* increases from 500 to 2000 mm, the energy absorption capacity of the structure decreases significantly, with *EA* values dropping from 4.61 to 0.415 kJ. This reduction is attributed to the exponential decay of shock wave intensity with distance, which diminishes both the peak pressure and duration of the blast load. The energy dissipation capacity of the panel exhibits a notable decrease with increasing *SOD*, primarily attributable to the reduction in blast impulse magnitude. This phenomenon stems from the characteristic attenuation of shock wave intensity with propagation distance, which simultaneously reduces both the peak pressure and duration of the applied blast load. At minimal *SOD* (500 mm), the panel undergoes extensive plastic deformation and core densification, facilitating superior energy absorption through material compaction. In contrast, larger *SOD* produce substantially attenuated loading conditions that generate diminished structural deformations and consequently alter the dominant energy dissipation mechanisms. These observations suggest that optimal panel design requires distinct strategies for different threat scenarios: close-range blast protection necessitates robust core configurations to withstand localized damage, while far-field blast resistance demands enhanced global stiffness to maintain structural integrity under reduced loading conditions.

### Effect of different panel thickness

**Fig. 14:**
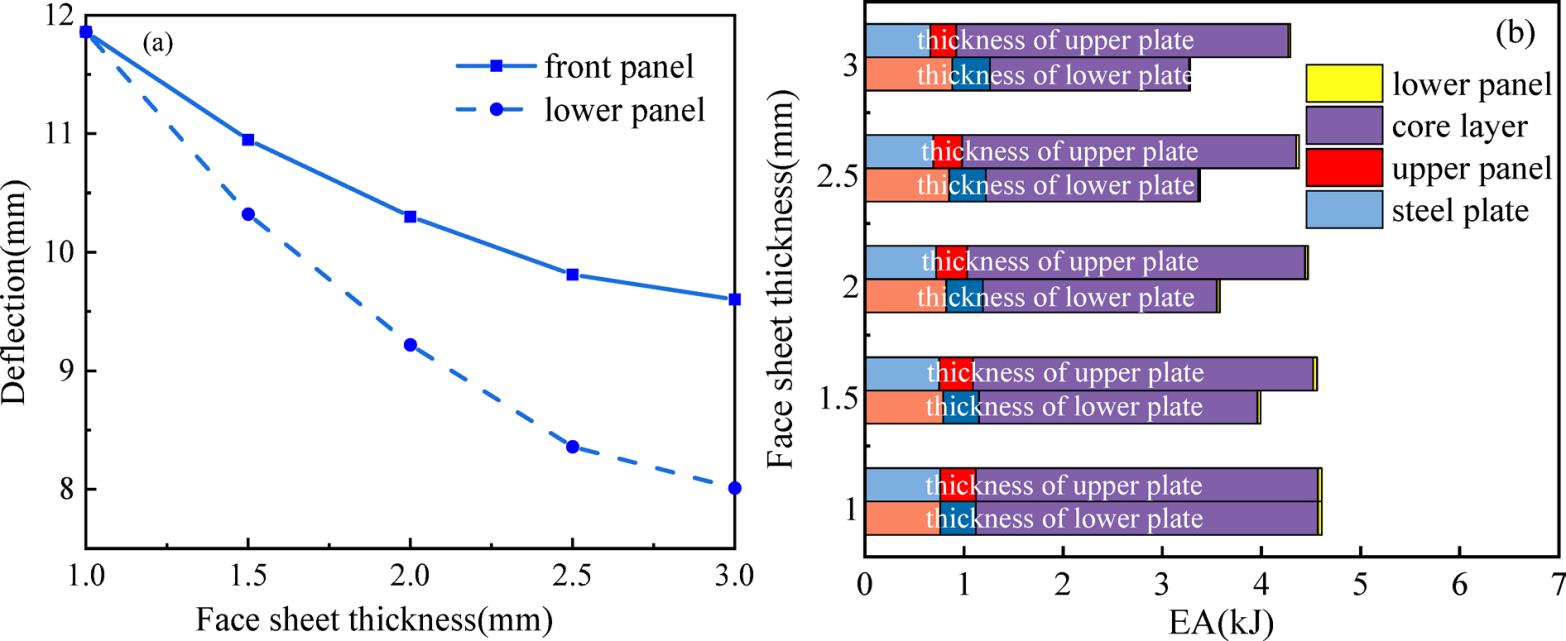
(**a**) *maxD* for sandwich panels with different panel thicknesses; (**b**) *EA*i distribution between sandwich panels with different panel thicknesses.

Table 3; Fig. 14a characterize the maximum displacements (*maxD*) exhibited by the sandwich composite system under varying upper (*t*u) and lower (*t*l) panel thickness configurations. The experimental results conclusively demonstrate that enhancing the panel thickness induces a marked decrease in the central deflection of the sandwich composite system. Thinner thickness results in lower overall stiffness of the sandwich panel. Hence, a larger thickness parameter corresponds to a smaller *maxD*. As *t*u increases from 1 to 3 mm, *maxD* decreases monotonically from 11.86 to 9.6 mm, indicating a 19.06% reduction. Similarly, when *t*l increases from 1 to 3 mm, it decreases by 32.46% from 11.86 to 8.01 mm. The variation in face-sheet thickness elicits differentiated structural behaviors in the sandwich composite system under blast loading. A thickened front face-sheet promotes more uniform blast load distribution, thereby expanding the compressive deformation zone within the core layer and enhancing energy absorption efficiency through optimized honeycomb collapse mechanisms, while concurrently mitigating peak central displacement. In contrast, increased back face-sheet thickness facilitates strain redistribution within the auxetic core, inducing preferential material flow toward the deformation focal point. This coordinated response mechanism effectively reduces overall structural displacement while simultaneously decreasing the system’s energy dissipation capacity. The observed thickness-dependent behavior demonstrates the critical role of face-sheet configuration in tailoring the blast-resistant performance of auxetic sandwich composites.

The energy dissipation capacity distribution of sandwich panels with different *t*u and *t*l also reflects this phenomenon, as illustrated in Fig. 14b and Table 3. As *t*u and *t*l increase, the *EA* decreases by 28.85% from 4.61 to 3.28 kJ and by 6.94% from 4.61 to 4.29 kJ. Augmenting the lower panel thickness induces a suppression of both peak centroidal displacement and global flexural deformation, thereby significantly diminishing the structural energy dissipation capacity. However, the energy dissipation mechanism of the composite system originates predominantly through compressive strain and lateral material redistribution within the core architecture, as opposed to global bending deformation, thereby maintaining consistent energy mitigation performance in the sandwich composite. Both *EA*c and *EA*l decrease as *t*u and *t*l increase. For instance, when *t*u = 1 mm, *EA*c and *EA*l are 3.45 kJ and 0.04 kJ, respectively, and for *t*u = 3 mm, *EA*c and *EA*l are 2.01 kJ and 0.01 kJ, respectively. Similarly, when *t*l = 1 mm, *EA*c and *EA*l are 3.45 kJ and 0.04 kJ, and for *t*l = 3 mm, *EA*c and *EA*l are 3.35 kJ and 0.02 kJ, respectively. Furthermore, as *t*l increases, the ratio of *EA*c to *EA* also increases, while the ratio of *EA*l to *EA* decreases.

### Effect of different gradient parameters

**Fig. 15:**
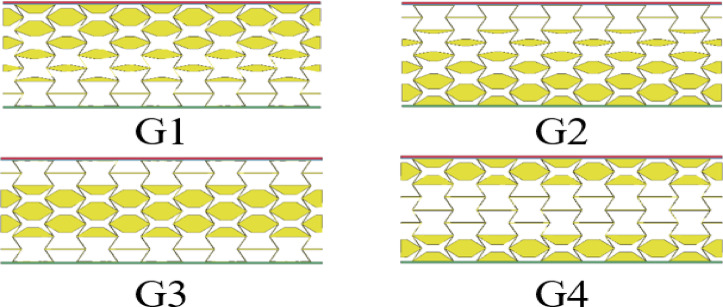
Schematic diagram of the gradient structure from G1 to G4.

**Fig. 16:**
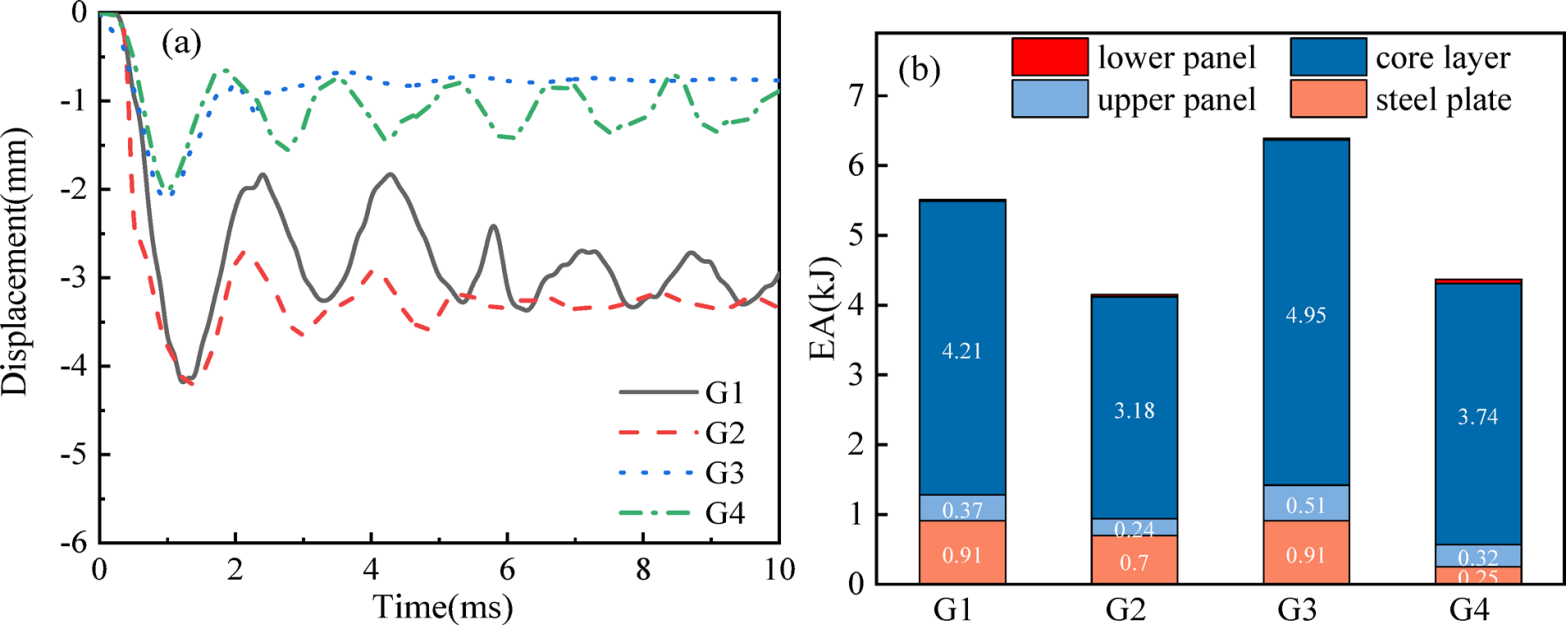
(**a**) *maxD* for sandwich plates with gradient parameters; (**b**) *EA*i distribution between sandwich plates with gradient parameters.

To systematically investigate the influence of gradient cellular architectures on blast resistance behavior, this research centers on through-thickness density variations within the core layer of sandwich composites. In this section, different unit stiffness is assigned to the sandwich structure to obtain various density gradients, denoted as *G*_1_ ~ *G*_4_. As shown in Fig. 15, *G*_1_ represents an asymptotic large model with stiffness of 100%, 80%, 60% and 40% for 1 ~ 4 layers. On the other hand, *G*_2_ represents an asymptotically small model with the layer stiffness arranged in the opposite order of *G*_1_. *G*_3_ is a model with an intermixed size of small-small layers, where the first and fourth layers have a stiffness of 100%, while the second and third layers have a VSF of 40%. *G*_4_, in contrast to *G*_3_, is a non-gradient model with layer stiffness arranged in the opposite order of *G*_3_, and it represents a size-interval model. Lastly, it is important to note that the density gradient remains consistent across all layers. Figure 16a presents the *maxD* and *EA* values of sandwich panels with different variable-stiffness factors. The findings demonstrate the critical influence of gradient parameter optimization on blast mitigation efficacy. For example, combinations *G*_1_ and *G*_2_ result in *maxD* values of 4.09 mm and 4.22 mm, respectively. Notably, a 3% reduction in *maxD* is achievable through strategic minimization of the VSF value at the blast-exposed interface. Moreover, *G*_3_ reduces *maxD* compared to *G*_4_, this additional material enhances the overall load-bearing capacity of the structure, subsequently leading to greater energy absorption.

For *EA*, the *G*_1_ facing layer shows the lowest cell stiffness core, resulting in an energy absorption value of 5.15 kJ. On the other hand, *G*_2_ shows the lowest cell stiffness core layer as the back-explosion surface layer, resulting in an energy absorption value of 4.15 kJ for the sandwich panel. Figure 16b illustrates the distribution of *EA*_i_ among sandwich panels with different gradient parameters. *EA*_c_ and *EA*_l_ exhibit progressive enhancement with layer-wise stiffness gradation from the blast-facing to the distal interface, mirroring the *EA* curve progression. Notably, in combination *G*_3_, the ratios of *EA*_c_ and *EA*_l_ to *EA* are 70.9% and 0.29% respectively. Similarly, in combination with *G*_4_, these ratios average at 85.5% and 1.4% respectively. The responses of EA and *maxD* for the four structures *G*_1_ to *G*_4_ are attributed to their unique deformation control mechanisms. The reduced stiffness of *G*_1_ enables the gradual energy dissipation in the rigid outer layer, transforming the elastic reflection of the inner layer into plastic collapse of the outer layer, thereby achieving a larger *EA*. The inverse gradient of *G*_2_ causes the soft layer to experience early stress concentration towards the explosion surface, reducing the redistribution of energy. The symmetrical design of *G*_3_ balances the reverse contraction of the flexible core and the rigid outer layer constraints, minimizing the maximum deformation. Therefore, gradient optimization controls the explosion resistance by adjusting the wave impedance, deformation sequence, and strain energy distribution.

## Conclusions

This research introduces a variable stiffness re-entrant (VSRE) core sandwich panel, developed through the variable stiffness factors (VSF) methodology. The blast resistance behavior of this composite structure was analyzed via finite element modeling and benchmarked against a re-entrant (RE)-cored counterpart. The validity of the finite element modeling method was verified by the results of field explosion tests. A computational framework based on the finite element method was established to systematically evaluate the influence of VSF values, re-entrant angles, panel thicknesses, stand-off distances, and gradient parameters on the blast mitigation performance of sandwich composite systems. Key findings derived from the computational modeling are synthesized below:

1. The VSRE core significantly outperforms conventional RE cores in blast mitigation, reducing maximum displacement (*maxD*) by 11.85% while increasing energy absorption (*EA*) by 14.39% at equal mass. With uniform thickness, performance improves further (81.11% *maxD* reduction, 27.0% *EA* increase), with over 70% of blast energy dissipated within the core. This enhanced performance stems from the VSRE core’s unique strain accommodation mechanism, where prioritized rib engagement in variable stiffness zones enhances interfacial stability, enabling superior energy dissipation and deformation resistance.
2. The study reveals that the blast resistance of VSRE core sandwich panels is highly dependent on key design parameters. Reducing the Variable Stiffness Factor (VSF) from 100 to 40% significantly improves performance, decreasing maximum displacement (*maxD*) by 430.1% while increasing energy absorption (*EA*) by 26.9%, as lower VSF values promote early cell densification and more efficient stress redistribution. The re-entrant angle (*θ*) also plays a crucial role, with larger angles (e.g., 60°) enhancing *EA* by 48.38% but increasing *maxD* by 18.15% due to greater material utilization and bending-dominated deformation. Stand-off distance (*SOD*) exhibits an inverse relationship with blast effects, where closer distances (500 mm) produce substantially higher *maxD* and *EA* compared to farther distances (2000 mm), reflecting the exponential decay of shock wave intensity. Additionally, increasing panel thickness, particularly the lower panel, reduces *maxD* by up to 32.46% by reinforcing structural support and promoting localized material densification, though it slightly diminishes overall energy absorption capacity. These findings underscore the importance of carefully balancing geometric and material parameters to optimize blast mitigation performance in VSRE sandwich panels.
3. The study demonstrates that core density gradient plays an important role in determining blast resistance performance. Specifically, *G*_1_ configuration shows a 3% reduction in maximum deflection compared to *G*_2_, suggesting that progressively decreasing stiffness from the blast-exposed surface improves structural resistance. Meanwhile, *G*_3_ exhibits better energy absorption and deformation control than *G*_4_, indicating that alternating high-low stiffness layers enhances overall performance. These findings clearly show the advantages of *G*_1_ and *G*_3_ configurations for blast mitigation applications. From a manufacturing perspective, producing gradient VSRE cores presents technical challenges that conventional laser cutting may not adequately address, especially for complex geometric variations in stiffness gradients. In this context, 3D printing emerges as a promising solution, offering the necessary precision for fabricating these sophisticated gradient VSF structures through its layer-by-layer additive manufacturing approach. Future work should expand the experimental validation to address these limitations more comprehensively.
4. The optimal blast mitigation design strategy is governed by the core architecture’s volumetric density ratio. For high relative densities, using a small VSF helps avoid complete core densification, effectively reducing lower panel deflection. On the other hand, reducing the relative density to lower levels requires increasing the re-entrant angle to consume more energy and enhance blast resistance.

## Data Availability

All data generated or analysed during this study are included in this published article.
